# Risk of Adverse Infant Outcomes Associated with Maternal Tuberculosis in a Low Burden Setting: A Population-Based Retrospective Cohort Study

**DOI:** 10.1155/2016/6413713

**Published:** 2016-02-16

**Authors:** Sylvia M. LaCourse, Sharon A. Greene, Elizabeth E. Dawson-Hahn, Stephen E. Hawes

**Affiliations:** ^1^Department of Medicine, Division of Allergy and Infectious Diseases, University of Washington, Seattle, WA 98104, USA; ^2^Department of Epidemiology, University of Washington, Seattle, WA 98195, USA; ^3^Department of Pediatrics, University of Washington, Seattle, WA 98121, USA

## Abstract

*Background*. Maternal tuberculosis (TB) may be associated with increased risk of adverse infant outcomes.* Study Design*. We examined the risk of low birth weight (LBW), small for gestational age (SGA), and preterm birth (<37 weeks) associated with maternal TB in a retrospective population-based Washington State cohort using linked infant birth certificate and maternal delivery hospitalization discharge records. We identified 134 women with births between 1987 and 2012 with TB-associated ICD-9 diagnosis codes at hospital delivery discharge and 536 randomly selected women without TB, frequency matched 4 : 1 on delivery year. Multinomial logistic regression analyses were performed to compare the risk of LBW, SGA, and preterm birth between infants born to mothers with and without TB.* Results*. Infants born to women with TB were 3.74 (aRR 95% CI 1.40–10.00) times as likely to be LBW and 1.96 (aRR 95% CI 0.91–4.22) as likely to be SGA compared to infants born to mothers without TB. Risk of prematurity was similar (aRR 1.01 95% CI 0.39–2.58).* Conclusion*. Maternal TB is associated with poor infant outcomes even in a low burden setting. A better understanding of the adverse infant outcomes associated with maternal TB, reflecting recent trends in US TB epidemiology, may inform potential targeted interventions in other low prevalence settings.

## 1. Introduction

Globally, tuberculosis (TB) is one of leading infectious causes of morbidity and mortality among women of childbearing age [1, 2]. Despite decreasing incidence of TB in the US since the 1990s, TB continues to disproportionately affect specific populations including immigrants [3]. In Washington State, the incidence of TB is similar to the US national average of 3.0 per 100,000; however, the proportion of TB cases occurring among foreign-born residents is greater (72.8% in Washington versus 60.0% in the US) [3, 4]. In 2013, women accounted for 41% of incident TB in Washington [4].

Pregnant and postpartum women may be at increased risk for TB potentially due to the physiologic, hormonal, and immunologic changes associated with pregnancy [5–7]. Additionally, pregnant women may not present with typical symptoms, which may delay diagnosis and lead to poor maternal outcomes [7–12]. Recent modelling estimates suggest that more than 200,000 TB cases occurred among pregnant women worldwide in 2011 [13]. Data regarding infant outcomes associated with maternal TB is conflicting, with some reporting increased adverse outcomes, intrauterine growth retardation, prematurity, small for gestational age, low birth weight, and death [14–19], while others show no increase in adverse outcomes [20].

The majority of studies evaluating adverse infant outcomes associated with maternal TB are case control studies within single facilities or small groups of hospitals, with very few population-based estimates conducted in low prevalence settings [10, 14, 18, 21–23]. A better understanding of the adverse infant outcomes associated with maternal TB reflecting recent trends in US TB epidemiology may inform potential targeted interventions in the US and other low prevalence settings. We conducted a population-based, retrospective cohort study to estimate the risk of low birth weight (LBW), small for gestational age (SGA), and prematurity among infants born to women with TB-associated hospital delivery discharge diagnoses in Washington State.

## 2. Materials and Methods

Women with singleton births in Washington State from 1987 to 2012 and their infants were identified using the Birth Events Records Database (BERD). The BERD database links more than 95% of Washington State birth certificate data to maternal and infant delivery hospitalization data from the statewide Comprehensive Hospital Abstract Reporting System (CHARS), including hospital discharge diagnosis codes by the International Classification of Diseases ninth revision (ICD-9) [24].

**Figure 1:**
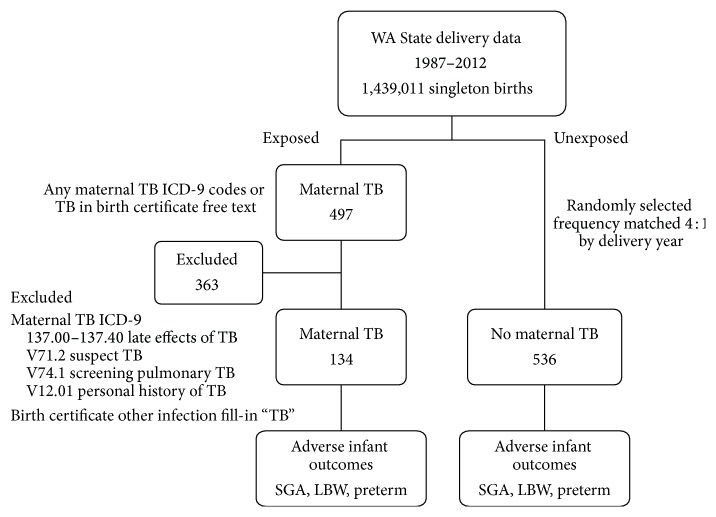
Study flow.

We initially identified all women delivering in Washington State between 1987 and 2012 with any TB-associated delivery diagnosis (ICD-9 codes 10.0–18.96, 647.30–647.34, V71.2, V74.1, and V12.01) or with TB noted on the birth certificate under “maternal infection other,” an open-ended data element on the birth certificate (Figure 1). We then excluded women with diagnoses that likely reflected a history of TB (as opposed to current TB), as well as TB suspect/screening (ICD-9 codes V71.2, V74.1, and V12.01), since we were specifically interested in the relationship between active maternal TB during pregnancy and delivery and its relationship to neonatal outcomes. Additionally, we excluded women identified through the open-ended “maternal infection other” on the birth certificate, as we were unable to discern whether it indicated the mother had latent TB infection or active TB from this data source. After these exclusions, there were 134 women in the final exposed maternal TB cohort (Figure 1). Supplemental Table 1 in Supplementary Material available online at http://dx.doi.org/10.1155/2016/6413713 shows the distribution of TB diagnoses identified from ICD-9 codes. The unexposed cohort was comprised of 536 randomly selected women without TB-associated diagnoses who delivered in Washington, frequency matched (4 : 1) on year of delivery (Figure 1).

The primary neonatal outcomes of SGA (weight < 10th percentile for age), LBW (<2,500 grams), and preterm birth (<37 weeks) were identified from information recorded on the birth certificate. Gestational age was coded as a composite variable using clinical estimate of gestational age or estimated using the last menstrual period (LMP) if the clinical estimate was missing. Weight for gestational age categories were derived from a population-based reference for Washington State [25]. We also evaluated secondary outcomes of infant mortality and congenital/infant TB from both birth and readmission data within the first two years of life from the BERD and CHARS databases.

Factors identified* a priori* as potential confounders in the relationship between maternal TB and poor neonatal outcomes included maternal age, education, race/ethnicity, parity, prenatal smoking, gestational diabetes, income (USD based on median census tract income), marital status, prenatal care (using the Adequacy of Prenatal Care Utilization index which classifies prenatal care based on date prenatal care was initiated and number of prenatal visits [26]), and maternal country of origin (grouped by World Bank gross national income index categories [27]). Maternal body mass index (BMI) was not available on the birth certificate before 2003. Weight gain during pregnancy was recorded on birth certificates beginning in 1988; however, prepregnancy weight was only available after 1992.

### 2.1. Statistical Analysis

We performed simple descriptive analyses comparing the frequency and distribution of the identified potential confounders between the exposure groups. Unadjusted risk estimates (uRR) for SGA, LBW, and preterm birth were compared between mothers with and without TB using multinomial logistic regression with the risk estimate converted to relative risk ratios (using mlogit, rrr command in STATA). Given our limited sample size and the high proportion of missing data for some factors, only potential confounders that changed the relative risk of our prespecified adverse infant outcomes by more than 10%, with less than <10% missing data, were included in our final multinomial logistic regression model. Adjusted relative risks (aRR) were calculated using multinomial logistic regression models adjusted for maternal age and income as continuous variables and parity and maternal country of origin as categorical variables with risk estimates converted to relative risk ratios. Relative risk estimates were reported with 95% confidence intervals (CI) and significance level was an alpha of 0.05. STATA (StataCorp, version 12, College Station, TX, USA) was used for all statistical analysis.

## 3. Results

**Figure 2:**
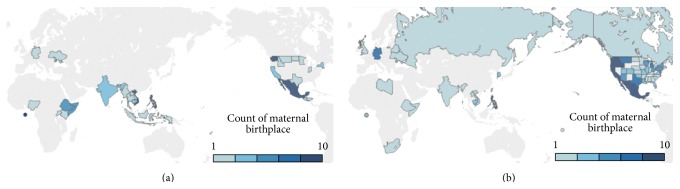
(a) Distribution of maternal birthplace for women with TB diagnosis. (b) Distribution of maternal birthplace for women without TB diagnosis.

Women with delivery hospitalization discharge TB-associated diagnosis were generally similar to the unexposed group with regard to maternal age and BMI (Table 1). Women with TB were less likely to be white (20.5 versus 79.4%), to graduate from high school (40.7 versus 82.9%), or to smoke during pregnancy (6.4 versus 15.8%), compared to women without TB. A larger proportion of women with TB were foreign-born (78.4 versus 16.8%), lived in urban areas (80.7 versus 74.7%), were single (36.6 versus 28.8%), had 2 or more previous pregnancies (35.1 versus 22.9%), and had gestational diabetes (6.0 versus 3.7%). Median census tract income among women with TB was lower than those without TB. Women with TB were more likely to have had either inadequate prenatal care (attending < 80% of the generally recommended number of prenatal visits) or were more likely to have experienced pregnancies with more intensive prenatal care visitation schedules (≥110%) than women without TB, who were most likely to have had the recommended prenatal care utilization. Among the 105 foreign-born women with TB, the majority were from Mexico (36.6%), followed by the Philippines (6.7%) and Vietnam (6.7%) (Figure 2).

Table 2 shows the unadjusted relative risks (uRR) for maternal TB exposure and adverse infant outcomes. Infants born to women with TB diagnosis at delivery were 2.64 times more likely to be LBW compared to infants born to women without TB (95% CI: 1.34–5.20). Similarly, infants born to women with TB were 1.95 times more likely to be SGA (95% CI: 1.11–3.41). Infants born to mothers with maternal TB had similar risk of preterm birth compared to those born to mothers without TB (RR 1.74, 95% CI: 0.89–3.43). After adjusting for maternal age, income, parity, and maternal country of origin, the risk of LBW remained increased (aRR 3.74 95% CI 1.40–10.00) for infants born to women with TB compared to those without TB. The risk of SGA remained similarly elevated (aRR 1.96 95% CI: 0.91–4.22) but no longer statistically significant. The risk of prematurity was attenuated but remained similar between infants born to women with and without TB (aRR 1.01 95% CI 0.39–2.58).

One infant born to a mother in our exposed cohort had a TB-associated diagnosis on readmission data within the first 24 months following birth; there were none in the unexposed cohort (data not shown). Two children in the exposed cohort and none in the unexposed cohort had an ICD-9 diagnosis code of “other congenital infections specific to the perinatal period” at their delivery admission or one of their readmissions during the first 24 months of life. This ICD-9 code includes TB, herpes simplex virus, listeriosis, malaria, and toxoplasmosis diagnoses. There was one fetal death reported in both the exposed and unexposed cohorts.

## 4. Discussion

In this population-based retrospective cohort study we found that maternal TB, as identified by maternal delivery discharge diagnosis ICD-9 codes, was significantly associated with low birth weight, with a trend toward an association with small for gestational age, but not prematurity, in a low TB-burden setting.

Our findings are similar to prior longitudinal studies of maternal TB and adverse infant outcomes. In a retrospective cohort study evaluating infant outcomes associated with TB in pregnancy in Taiwan (a high TB burden setting), Lin et al. reported that infants born to mothers with TB were 1.35 (95% CI: 1.01–1.81) times as likely to be LBW and 1.22 (95% CI: 1.00–1.49) times as likely to be SGA compared to infants born to mothers without TB [17]. Similar to the current study, no association was found with prematurity (RR 0.97, 95% CI: 0.72–1.30). Lower mean birth weights and increased risk of LBW among infants born to mothers with TB compared to those born to others without TB have been reported in a retrospective single-hospital based cohort study in Mexico (mean birth weight 2,859 versus 3,099 grams; RR of LBW 2.2 (95% CI 1.1–4.9)) [16]. Similarly, a small case control study in three inner-city hospitals in the UK found that 24 infants born to women with TB had a mean birth weight of 2,735 grams, which was significantly lower than the mean weight of 3,135 grams (*p* = 0.03) of infants of mothers without TB [18]. Finally, in an obstetric specialty clinic in India, infants born to women with TB were 2.1 times as likely to be born LBW (95% CI: 1.4–3.1) and 2.6 times as likely to be born SGA (95% CI: 1.4–4.6) [19].

We identified very few cases of infant TB in our study. Although we do not have specific maternal TB diagnosis and treatment information, it is likely that, in Washington State, once the diagnosis of maternal TB is made, the mother would have been initiated on therapy, significantly decreasing the risk of transmission of TB to the infant. Conversely, an infant TB diagnosis may have been made in the outpatient setting which would not have been captured by the inpatient diagnoses codes unless the infant was hospitalized. However, in our setting, gastric aspirates (one of the more common means for making infant TB diagnosis) often require hospitalization. Without medical record confirmation, it is difficult to assess congenital TB since the ICD-9 code used to identify congenital TB is used for a multitude of congenital infections in the perinatal period including TB, herpes simplex, listeriosis, and toxoplasmosis (however, in our study only two children were identified with this composite code).

The few prevalence estimates of TB in pregnancy in low burden settings vary widely and are primarily facility or hospital based [7, 10, 14, 18, 21–23]. Maternal TB is thought to be uncommon in the US; however, pregnancy status is not routinely collected or reported for either state or national TB estimates, and there is a gap in the literature for population-based studies of the risks of adverse neonatal outcomes in low TB prevalence settings. Our study is the first population-based study to our knowledge that evaluates the risk of adverse infant outcomes associated with maternal TB reflecting recent US TB epidemiologic trends while accounting for maternal country of origin. This investigation contributes to the understanding that adverse infant outcomes may be associated with maternal TB even in a low burden setting and can be used to inform targeted interventions in Washington, in the US, or in other low prevalence settings.

In the present study, TB in pregnancy is higher among women born outside of the United States and among women with lower educational attainment and income. Clinicians should be aware of at risk populations and the importance of diagnosing and treating TB during pregnancy. The US Centers for Disease Control and Prevention recommends that TB treatment should be initiated during pregnancy because the risk of untreated TB is a greater hazard for both the mother and infant [28]. Although we do not know the TB screening status of the patients in this study, pregnancy has been associated with missed opportunities for TB prevention [7, 29]. Universal screening of high risk obstetric patients for latent TB could identify a high proportion of women who are eligible for latent TB therapy [30]. Cost-effectiveness modelling estimates antenatal provision of isoniazid preventive therapy is potentially less costly with reduced maternal TB-associated case-fatality compared to postnatal TB prevention strategies [31]. Our findings reinforce the need for intrapartum support of mothers diagnosed with TB and sustained public health surveillance of maternal TB to minimize the likelihood of delivering LBW and potentially SGA infants.

Strengths of our study include using a large population-based cohort spanning 25 years. Utilizing an electronic repository of maternally linked birth outcome data allowed us to estimate the risk of adverse infant outcomes associated with maternal TB diagnosis. Given that the TB incidence in Washington State is similar to the US incidence in general, Washington State risk estimates of neonatal outcomes may also reflect current US TB epidemiology and aid in the characterization of poor infant outcomes associated with maternal TB in low burden settings.

There were some limitations with our approach. Ascertainment of both our exposure (maternal TB) and outcomes of interest (SGA, LBW, and prematurity) were based on data from the Washington State Birth Events Records Database (BERD) and Comprehensive Hospital Abstract Reporting System (CHARS). As with any large public database system, there may be issues with either misreporting or miscoding of information. In an evaluation of Washington State birth certificate and hospital discharge data, there was substantial underreporting of maternal conditional and pregnancy complications on birth certificates; however, hospital discharge data was found to be more accurate [32]. The combination of birth certificate and hospital discharge data, in the linked BERD and CHARS database, yields higher “true positives” than either data source alone [32]. The number of women in Washington with a TB diagnosis recorded on hospital discharge records was low; we cannot exclude the possibility of underdiagnosis or underreporting of maternal TB. Additionally, women are not routinely tested for TB during prenatal care visits and a woman who is asymptomatic or has atypical symptoms for TB may not be diagnosed [33]. There is no specific data element on the birth certificate for TB; rather we are relying on clinicians to report TB diagnosis in the hospital discharge records. The clinician may be unaware of the mother's TB status or may choose not to report TB on a discharge record for delivery. Our study is also limited by the lack of specific clinical information regarding date or method of TB diagnosis, as well as treatment. The risk of developing TB is higher among people living with HIV [34]. Washington birth certificate captures information on maternal HIV infection; but this sensitive data was not available for analysis. However, in Washington, the prevalence of HIV among new TB patients is low at 2.6% [35]. Therefore, we do not anticipate our lack of HIV data to alter our risk estimates.

In our cohort, the majority of mothers with TB were foreign-born, consistent with trends in both Washington State and the US in general. Despite a significant decrease in TB incidence in the general US population since 1993, the decrease among foreign-born persons has been much smaller, with the case rate among foreign-born persons approximately 13 times higher than among US-born (15.6 versus 1.2 per 100,000) [35]. The increased risk of adverse infant outcomes associated with maternal TB in our study could reflect an increased risk of SGA and prematurity of infants born to foreign-born mothers. In order to address this potential confounder directly, we adjusted our risk estimates of adverse infant outcomes by maternal country of origin (as categorized by World Bank gross national income index). However, residual confounding may remain. Although maternal country of origin may be an important driver of poor infant outcomes, there is a growing body of literature suggesting that infants born to foreign-born mothers may in fact have better birth outcomes compared to ethnically similar US-born mothers [36–40]. In the few comparable studies in the literature (i.e., Taiwan [17] and India [19]) the association between maternal TB and adverse infant outcomes was similar, suggesting that the difference between foreign-born and US-born mothers in our study is less likely to be influencing our results regarding neonatal outcomes.

In the current study, we adjusted for a number of factors, including maternal age, income, parity, and maternal country of origin. Missing data for a number of factors (maternal education, BMI, and weight gain during pregnancy) was substantial, limiting the ability to completely adjust for these factors. Therefore, it is possible that the relationship between maternal TB status and adverse infant outcomes in our study may be due to unmeasured or residual confounding. Future studies using larger population-based estimates may be needed to assess the importance of the factors more fully.

## 5. Conclusion

Our study found a substantially increased risk of LBW and a nonsignificant trend towards increased risk of SGA among infants born to mothers with TB diagnosis even in a low burden setting, suggesting that maternal TB remains an important risk factor for adverse infant outcomes. This highlights the importance of close clinical follow-up of pregnant women with TB and their infants. Women from high-incidence countries may warrant increased vigilance for latent TB screening and treatment to prevent adverse maternal and infant outcomes associated with maternal TB.

Further research regarding both the risk of TB in pregnancy and the risk of neonatal adverse outcomes associated with maternal TB is warranted, including detailed case ascertainment. Efforts could be aided by the addition of pregnancy status to TB surveillance efforts. A better understanding of mothers at risk for TB and the adverse infant outcomes associated with maternal TB may inform potential targeted interventions in other low prevalence settings.

## Supplementary Material

Tuberculosis ICD-9 diagnosis codes used to identify the maternal TB exposed cohort delivering in Washington State, 1987-2012.

## Figures and Tables

**Table 1:** Characteristics of women with and without tuberculosis-associated ICD-9 diagnosis during delivery hospitalization, Washington State, 1987–2012.

Maternal characteristic	% Missing	Maternal TB (*N* = 134)	No maternal TB (*N* = 536)
		*n* ^*∗*^ (%)	*n* ^*∗*^ (%)
Age (years)	0		
<20		14 (10.5)	54 (10.1)
20–29		80 (59.7)	294 (54.9)
≥30		40 (29.9)	188 (35.1)
Median age (IQR)		26 (22–31)	27 (23–32)
Ethnicity	2.2		
White		27 (20.5)	415 (79.4)
Black		14 (10.6)	13 (2.5)
Asian		33 (25.0)	28 (5.4)
Hispanic		52 (39.4)	47 (9.0)
Native American		6 (4.6)	18 (3.4)
Other non-white		0 (0)	2 (0.4)
Education	34.8		
<High school graduate		51 (59.3)	60 (17.1)
High school graduate		35 (40.7)	291 (82.9)
Median year of education (IQR)		10 (9–12)	13 (12–16)
Urban residence^§^	10.3	96 (80.7)	360 (74.7)
Income (dollars)^‡^	8.1		
<35,000		63 (52.5)	209 (42.1)
35,000–49,999		48 (40.0)	174 (35.1)
≥50,000		9 (7.5)	113 (22.8)
Median income (in thousands) (IQR)		34 (26–40)	37 (31–48)
Single marital status	0.2	49 (36.6)	154 (28.8)
Foreign born	0	105 (78.4)	90 (16.8)
Maternal country of origin^¶^	1.5		
Low-income		53 (40.5)	235 (44.4)
Middle-income		32 (24.4)	173 (32.7)
High-income		46 (35.1)	121 (22.9)
Parity	1.5		
0		53 (40.5)	235 (44.4)
1		32 (24.4)	173 (32.7)
≥2		46 (35.1)	121 (22.9)
Prenatal care utilization^†^	11.2		
<80% PNC visits		53 (46.9)	157 (32.6)
80–109% PNC visits		36 (31.9)	248 (51.5)
≥110% PNC visits		24 (21.2)	77 (16.0)
Body mass index (BMI)^#^	72.5		
BMI < 18.5 underweight		0 (0)	4 (2.6)
BMI 18.5–24.9 normal		18 (56.3)	76 (50.0)
BMI 25.0–29.9 overweight		8 (25.0)	38 (25.0)
BMI ≥ 30.0 obese class I–III		6 (18.8)	34 (22.4)
Median BMI (IQR)		23.6 (20.7–27.9)	24.8 (22.2–29.4)
Weight gain in pregnancy (lbs)^*∗∗*^	28.7		
Loss or no gain		0 (0)	2 (0.5)
1–9.9		6 (6.7)	12 (3.1)
10–19.9		17 (18.9)	40 (10.3)
20–39.9		48 (53.3)	205 (52.8)
>40		19 (21.1)	129 (33.3)
Median pregnancy weight gain (IQR)		26 (19–37)	33 (25–40)
Gestational diabetes	0	8 (6.0)	20 (3.7)
Smoked during pregnancy	5.5	8 (6.4)	80 (15.8)

^*∗*^Numbers may not add up to totals because of missing data.

^§^Estimated by linking birth certificates to census tract records from U.S. Census Bureau 2000.

^**‡**^Estimated by linking birth certificates to census tract records from U.S. Census Bureau 2000, median income per census tract.

^¶^Per World Bank gross national income index classifications. Lower-middle and upper-middle income countries are categorized together as middle-income.

^†^Kotelchuck index classifies prenatal care based on birth certificate data on date prenatal care was initiated and number of prenatal visits.

^#^BMI was not available on the birth certificate before 2003.

^*∗∗*^Weight gain during pregnancy was recorded on birth certificates from 1988 on, however pre-pregnancy weight was only available after 1992.

**Table 2:** Risk of small for gestational age, low birth weight, and prematurity for infants born to women with tuberculosis-associated ICD-9 diagnosis during delivery hospitalization, Washington State, 1987–2012.

Outcome	Maternal TB (*N* = 134)	No maternal TB (*N* = 536)	Unadjusted RR		Adjusted RR^§^	
	*n* (%)	*n* (%)	uRR	(95% CI)	aRR	(95% CI)
Birth weight (grams)						
LBW (<2,500)	15 (11.9)	24 (4.5)	**2.64**	(1.34–5.20)	**3.74**	(1.40–10.00)
Typical (2,500–3,999)	105 (78.4)	443 (82.7)	1.00	Referent	1.00	Referent
Macrosomia (≥4,000)	14 (10.5)	69 (12.9)	0.86	(0.46–1.58)	1.39	(0.59–3.30)
Weight for gestational age^*∗*^						
SGA (<10%)	21 (15.8)	47 (8.9)	**1.95**	(1.11–3.41)	1.96	(0.91–4.22)
Typical (10–90%)	100 (75.2)	436 (82.4)	1.00	Referent	1.00	Referent
LGA (>90%)	12 (9.0)	46 (8.7)	1.13	(0.58–2.23)	1.69	(0.63–4.55)
Gestational age (weeks)^*∗*^						
<37 (preterm)	13 (9.8)	32 (6.0)	1.74	(0.89–3.43)	1.01	(0.39–2.58)
37–41	112 (84.9)	481 (90.9)	1.00	Referent	1.00	Referent
≥42 (postterm)	7 (5.3)	16 (3.0)	1.91	(0.54–6.74)	1.91	(0.53–6.75)

^*∗*^Numbers may not add up to totals due to missing data.

^§^Model adjusted for maternal age, income, parity, and maternal country of origin World Bank gross national income index category.

## References

[1] Mathad J. S., Gupta A. (2012). Tuberculosis in pregnant and postpartum women: epidemiology, management, and research gaps. *Clinical Infectious Diseases*.

[2] Getahun H., Sculier D., Sismanidis C., Grzemska M., Raviglione M. (2012). Prevention, diagnosis, and treatment of tuberculosis in children and mothers: evidence for action for maternal, neonatal, and child health services. *The Journal of Infectious Diseases*.

[3] Centers for Disease Control and Prevention TB Incidence in the United States, 1953–2013. http://www.cdc.gov/tb/statistics/tbcases.htm.

[4] Washington State Department of Health (2014). *Tuberculosis: Data and Reports*.

[5] Singh N., Perfect J. R. (2007). Immune reconstitution syndrome and exacerbation of infections after pregnancy. *Clinical Infectious Diseases*.

[6] Zenner D., Kruijshaar M. E., Andrews N., Abubakar I. (2012). Risk of tuberculosis in pregnancy: a national, primary care-based cohort and self-controlled case series study. *American Journal of Respiratory and Critical Care Medicine*.

[7] Jana N., Barik S., Arora N., Singh A. K. (2012). Tuberculosis in pregnancy: the challenges for South Asian countries. *Journal of Obstetrics and Gynaecology Research*.

[8] Gupta A., Chandrasekhar A., Gupte N. (2011). Symptom screening among HIV-infected pregnant women is acceptable and has high negative predictive value for active tuberculosis. *Clinical Infectious Diseases*.

[9] Hoffmann C. J., Variava E., Rakgokong M. (2013). High prevalence of pulmonary tuberculosis but low sensitivity of symptom screening among HIV-infected pregnant women in South Africa. *PLoS ONE*.

[10] Llewelyn M., Cropley I., Wilkinson R. J., Davidson R. N. (2000). Tuberculosis diagnosed during pregnancy: a prospective study from London. *Thorax*.

[11] Jana N., Vasishta K., Saha S. C., Ghosh K. (1999). Obstetrical outcomes among women with extrapulmonary tuberculosis. *The New England Journal of Medicine*.

[12] Bishara H., Lidji M., Vinitsky O., Weiler-Ravell D. (2014). Indolent pneumonia in a pregnant recent immigrant from Ethiopia: think TB. *Primary Care Respiratory Journal*.

[13] Sugarman J., Colvin C., Moran A. C., Oxlade O. (2014). Tuberculosis in pregnancy: an estimate of the global burden of disease. *The Lancet Global Health*.

[14] Kothari A., Mahadevan N., Girling J. (2006). Tuberculosis and pregnancy—results of a study in a high prevalence area in London. *European Journal of Obstetrics Gynecology and Reproductive Biology*.

[15] Pillay T., Khan M., Moodley J., Adhikari M., Coovadia H. (2004). Perinatal tuberculosis and HIV-1: considerations for resource-limited settings. *The Lancet Infectious Diseases*.

[16] Figueroa-Damian R., Arredondo-Garcia J. L. (2001). Neonatal outcome of children born to women with tuberculosis. *Archives of Medical Research*.

[17] Lin H.-C., Lin H.-C., Chen S.-F. (2010). Increased risk of low birthweight and small for gestational age infants among women with tuberculosis. *BJOG*.

[18] Asuquo B., Vellore A. D., Walters G., Manney S., Mignini L., Kunst H. (2012). A case-control study of the risk of adverse perinatal outcomes due to tuberculosis during pregnancy. *Journal of Obstetrics & Gynaecology*.

[19] Jana N., Vasishta K., Jindal S. K., Khunnu B., Ghosh K. (1994). Perinatal outcome in pregnancies complicated by pulmonary tuberculosis. *International Journal of Gynecology and Obstetrics*.

[20] Tripathy S. N., Tripathy S. N. (2003). Tuberculosis and pregnancy. *International Journal of Gynecology and Obstetrics*.

[21] Centers for Disease Control and Prevention (1993). Tuberculosis among pregnant women—New York city, 1985–1992. *Morbidity and Mortality Weekly Report (MMWR)*.

[22] Good J. T., Iseman M. D., Davidson P. T., Lakshminarayan S., Sahn S. A. (1981). Tuberculosis in association with pregnancy. *American Journal of Obstetrics and Gynecology*.

[23] Schwartz N., Wagner S. A., Keeler S. M., Mierlak J., Seubert D. E., Caughey A. B. (2009). Universal tuberculosis screening in pregnancy. *American Journal of Perinatology*.

[24] Herman A. A., McCarthy B. J., Bakewell J. M. (1997). Data linkage methods used in maternally-linked birth and infant death surveillance data sets from the United States (Georgia, Missouri, Utah and Washington state), Israel, Norway, Scotland and Western Australia. *Paediatric and Perinatal Epidemiology*.

[25] Lipsky S., Easterling T. R., Holt V. L., Critchlow C. W. (2005). Detecting small for gestational age infants: the development of a population-based reference for Washington State. *American Journal of Perinatology*.

[26] Kotelchuck M. (1994). An evaluation of the kessner adequacy of prenatal care index and a proposed adequacy of prenatal care utilization index. *American Journal of Public Health*.

[27] The World Bank Group (2015). *Country and Lending Groups*.

[28] Centers for Disease Control and Prevention (2003). Treatment of tuberculosis. *MMWR Recommendations and Reports*.

[29] Slopen M. E., Laraque F., Piatek A. S., Ahuja S. D. (2011). Missed opportunities for tuberculosis prevention in New York City, 2003. *Journal of Public Health Management and Practice*.

[30] Mamishi S., Pourakbari B., Teymuri M. (2014). Diagnostic accuracy of IL-2 for the diagnosis of latent tuberculosis: a systematic review and meta-analysis. *European Journal of Clinical Microbiology and Infectious Diseases*.

[31] Boggess K. A., Myers E. R., Hamilton C. D. (2000). Antepartum or postpartum isoniazid treatment of latent tuberculosis infection. *Obstetrics and Gynecology*.

[32] Lydon-Rochelle M. T., Holt V. L., Cárdenas V. (2005). The reporting of pre-existing maternal medical conditions and complications of pregnancy on birth certificates and in hospital discharge data. *American Journal of Obstetrics & Gynecology*.

[33] Carter E. J., Mates S. (1994). Tuberculosis during pregnancy: the Rhode Island experience, 1987 to 1991. *Chest*.

[34] World Health Organization Global Tuberculosis Report 2014. http://www.who.int/tb/publications/global_report/en/.

[35] Centers for Disease Control (2014). *Reported Tuberculosis in the United States, 2014*.

[36] Margerison-Zilko C. (2014). The contribution of maternal birth cohort to term small for gestational age in the United States 1989–2010: an age, period, and cohort analysis. *Paediatric and Perinatal Epidemiology*.

[37] Elo I. T., Vang Z., Culhane J. F. (2014). Variation in birth outcomes by mother's country of birth among non-Hispanic black women in the United States. *Maternal and Child Health Journal*.

[38] Osypuk T. L., Bates L. M., Acevedo-Garcia D. (2010). Another Mexican birthweight paradox? The role of residential enclaves and neighborhood poverty in the birthweight of Mexican-origin infants. *Social Science and Medicine*.

[39] Singh G. K., Stella M. Y. (1996). Adverse pregnancy outcomes: differences between US- and foreign-born women in major US racial and ethnic groups. *American Journal of Public Health*.

[40] Qin C., Gould J. B. (2010). Maternal nativity status and birth outcomes in Asian immigrants. *Journal of Immigrant and Minority Health*.

