# Supplementary material for: Risk of Adverse Infant Outcomes Associated with Maternal Tuberculosis in a Low Burden Setting: A Population-Based Retrospective Cohort Study
Source: Infect Dis Obstet Gynecol. 2016 Feb 16;2016:6413713. doi: 10.1155/2016/6413713 (PMC4771913; doi:10.1155/2016/6413713)
Supplement: Supplementary file 1 — Tuberculosis ICD-9 diagnosis codes used to identify the maternal TB exposed cohort delivering in Washington State, 1987-2012. [file 6413713.f1.pdf]

Supplemental Table 1. Tuberculosis diagnoses used to identify the maternal TB exposed cohort delivering in Washington State, 1987-2012

ICD 9 code	Description	Maternal TB N=134 n	(%)
10.00-18.99	Specific TB associated ICD 9 codes		
11.90-11.92	Pulmonary	17	(12.7)
18.00-18.99	Disseminated	1	(0.8)
13.00-17.99	Extra-pulmonary	6	(4.5)
647.30-647.34	TB in pregnancy	110	(82.1)
